# Wearable Two-Dimensional Nanomaterial-Based Flexible Sensors for Blood Pressure Monitoring: A Review

**DOI:** 10.3390/nano13050852

**Published:** 2023-02-24

**Authors:** Siti Nor Ashikin Ismail, Nazrul Anuar Nayan, Muhammad Aniq Shazni Mohammad Haniff, Rosmina Jaafar, Zazilah May

**Affiliations:** 1 Department of Electrical, Electronic and Systems Engineering, Universiti Kebangsaan Malaysia, Bangi 43600 UKM, Selangor, Malaysia; 2 Institute Islam Hadhari, Universiti Kebangsaan Malaysia, Bangi 43600 UKM, Selangor, Malaysia; 3 Institute of Microengineering and Nanoelectronics, Universiti Kebangsaan Malaysia, Bangi 43600 UKM, Selangor, Malaysia; 4 Electrical and Electronic Engineering Department, Universiti Teknologi Petronas, Seri Iskandar 32610, Perak, Malaysia

**Keywords:** flexible, sensors, blood pressure, 2D nanomaterials, wearable

## Abstract

Flexible sensors have been extensively employed in wearable technologies for physiological monitoring given the technological advancement in recent years. Conventional sensors made of silicon or glass substrates may be limited by their rigid structures, bulkiness, and incapability for continuous monitoring of vital signs, such as blood pressure (BP). Two-dimensional (2D) nanomaterials have received considerable attention in the fabrication of flexible sensors due to their large surface-area-to-volume ratio, high electrical conductivity, cost effectiveness, flexibility, and light weight. This review discusses the transduction mechanisms, namely, piezoelectric, capacitive, piezoresistive, and triboelectric, of flexible sensors. Several 2D nanomaterials used as sensing elements for flexible BP sensors are reviewed in terms of their mechanisms, materials, and sensing performance. Previous works on wearable BP sensors are presented, including epidermal patches, electronic tattoos, and commercialized BP patches. Finally, the challenges and future outlook of this emerging technology are addressed for non-invasive and continuous BP monitoring.

## 1. Introduction

Physiological monitoring offers deep understanding to evaluate the health state of an individual [1]. In recent years, several attempts have been made to modify existing sensors for developing wearable point-of-care systems that are suitable for personalized health monitoring [2,3]. The widespread use of wearable sensors in healthcare have heightened the need for non-invasive, continuous, and real-time monitoring of vital signs, such as blood pressure (BP). Wearable technologies play a significant role in improving human life quality through accurate disease prediction, timely treatment, reduced medical burden, and improved longevity [4,5,6,7,8]. A rapid development of wearable devices has occurred since a decade ago; these devices are not limited to smartwatches [9] and fitness bands [10], and other sensing devices, such as skin patches [11,12,13] and electronic tattoos (e-tattoo) [14,15,16], are gaining increased research attention because they are more appealing to users. Previous works demonstrated how flexible sensors enable a wide pressure detection range [17,18,19].

The advent of flexible materials has led to the plethora of studies on two-dimensional (2D) nanomaterials as promising candidates for the development of sensing elements in flexible sensors. The extensive exploitation of these materials, particularly since the successful exploration of graphene (Gr) by Novoselov et al. [20] in 2004, has inspired the discovery of other similar sheet-like structures, such as transition metal dichalcogenides (TMDs) and transition metal carbides and nitrides (MXenes).

In contrast to other candidates, such as silver (Ag) and copper (Cu), 2D nanomaterials have a number of attractive features, such as larger surface-area-to-volume ratio, higher electrical conductivity, cost effectiveness, flexibility, and lighter weight [21,22,23,24,25,26,27], which confer sensors with excellent wearable sensing performance. Further, conventional sensors are mainly fabricated based on silicone (Si) or glass substrates, making them rigid, bulky, and incapable for physiological monitoring [28,29,30], which in turn degrade the sensor performance. The two tools currently used for BP measurement are the arterial catheter and the sphygmomanometer. The former is the gold standard in BP monitoring and mainly employed to measure continuous and accurate BP readings of critical patients [31]. A major problem of this modality is that patients often feel pain from invasive catheter insertion [32] and trained personnel are required to perform the measurement [33]. The latter is non-invasive, more practical, and has expanded usability outside clinical settings. Although various cuff-based BP monitors are available on the market, their use has been associated with certain drawbacks, such as intermittent measurements, calibration issues, motion sensitivity, and whitecoat effect [34,35,36,37], leading to inaccurate detection. Current studies show an increasing trend of developing non-invasive and cuffless BP sensors by using photoplethysmogram and electrocardiogram (ECG); however, these techniques are prone to produce motion artifacts, BP variations across devices, and shallow penetration depth into the deep artery [38,39,40].

The continuous development of 2D wearable nanomaterial-based technologies is needed to overcome the aforementioned challenges and offer a non-invasive and continuous BP monitoring system with great skin conformability, biocompatibility, improved efficiency, and accurate results. In comparison with other review studies [41,42], the present work mainly focuses on the recent advances of wearable flexible sensors based on 2D nanomaterials for BP monitoring in a non-invasive and continuous manner.

**Figure 1:**
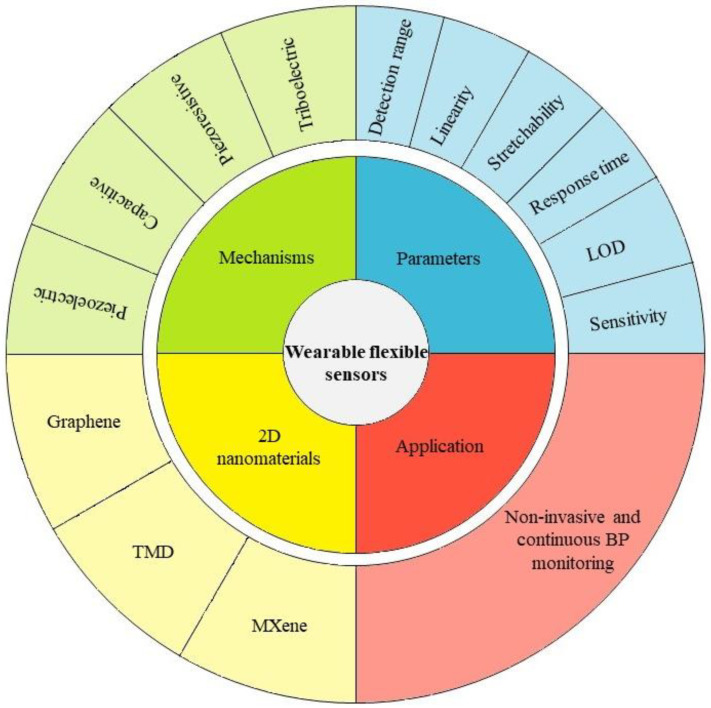
Overview of the paper.

Figure 1 illustrates the overview of the paper, which is divided into seven sections. Section 2 describes the mechanisms of flexible sensors, and Section 3 introduces several parameters of sensing performance. Section 4 discusses the 2D nanomaterials frequently used to fabricate flexible sensors and presents a comparison between 2D nanomaterials explored as sensing materials for flexible sensors and their respective performance. Section 5 reports previously published works of wearable BP sensors. Section 6 addresses the challenges faced by current techniques and future outlooks on wearable sensing implementation. Section 7 summarizes the overview of the paper and ends with a conclusion.

## 2. Transduction Mechanisms

Physiological monitoring can be performed using flexible sensors to monitor vital signs in a non-invasive and continuous manner. BP monitoring is conducted by placing these sensors on the skin, and electrical signals are generated when subjected to a mechanical stimulus. In this section, several transduction mechanisms, including piezoelectric, capacitive, piezoresistive, and triboelectric, are described.

### 2.1. Piezoelectric Mechanism

The piezoelectric effect was discovered by the Curie brothers in 1880 [43]. The study of piezoelectric has grown significantly since the employment of quartz crystals in sonar [44]. It was then replaced by inorganic piezoelectric materials, such as barium titanate and lead zirconate titanate (PZT), which exhibit distinguished piezoelectric characteristics [45]. Recent studies have shown that organic materials made from polymers, such as polyvinylidene fluoride [46], are preferred in biomedical devices due to their flexibility, light weight, and capability to generate energy [47]. The piezoelectric mechanism has been widely used in different technologies, such as sensors and actuators [48,49,50,51,52,53,54].

Piezoelectric sensor is based on electrical signals generated when deformation occurs on non-centrosymmetric materials. When these materials experience stress, the crystal structure becomes dislocated, causing the accumulation of positive and negative charges across the faces. As a result, net polarization is formed throughout the crystal structure. Significant potential difference is observed when current is applied to the sensing system.

The advantage of using a piezoelectric sensor is that it can measure dynamic signals. Moreover, it performs best in terms of sensitivity, fast response time, and low power consumption. The piezoelectric behavior of 2D nanomaterials has gained considerable interest since it was reported in 2014 [55]. However, the main disadvantages of this sensor are its inability to monitor static signals, low sensitivity for detection of low pressure, and use of extremely harmful lead-based materials.

### 2.2. Capacitive Mechanism

The capacitive effect of an Si-based sensor was first studied by Michael Faraday in 1831 [56]. The capacitive transduction mechanism differs from piezoelectric and piezoresistive mechanisms in that no pressure is applied to introduce deformation, but it instead involves the contact between two plates. The parallel-plate capacitor is a typical arrangement of a capacitive sensor, wherein two plates are separated from each other by a dielectric layer. A Si-based organic polymer known as polydimethylsilixone (PDMS) has been widely investigated as a dielectric layer in capacitive sensors due to its great flexibility, biocompatibility, and low toxicity [57,58]. A reduction in separation distance between plates leads to a change in capacitance [59], as shown in Equation (1):

(1) C=εA/d,

where C is the capacitance, ε is the dielectric constant, A is the area of the plate, and d is the separation distance between plates.

Capacitive sensors have a number of advantages over piezoelectric-based sensors in terms of lower power consumption, wider detection range, and higher sensitivity for the detection of low pressure. However, this sensor has slow response time, an expensive fabrication procedure, and power source dependency. The sensitivity of capacitive sensors can be increased by several recommended methods, such as lowering the dielectric layer resistance, finetuning the above parameters, and miniaturizing on-chip integrated circuits [60].

### 2.3. Piezoresistive Mechanism

The piezoresistive effect is not a recent discovery, given that its first experimental demonstration was carried out in 1954 by Smith [61]. The resistivity of a material was identified as the main factor contributing to the change in resistance [62]. The contact area of dielectric materials is expanded by imposing mechanical stimulation, thus allowing the formation of more conductive channels [59]. This phenomenon changes the material resistivity and causes variations in electrical signals [63]. Gr and carbon nanotubes are frequently exploited in the development of piezoresistive flexible sensors. When the applied stress is removed, the resistance returns to its original state. Equation (2) represents the relative change in the resistance of piezoresistive sensors:

(2) $$\Delta R/R_{0}=\left(1+2v\right)\varepsilon +\Delta \rho /\rho ,\;$$

where $\Delta R/R_{0}$ is the change of resistance, v is the Poisson’s ratio, ε is the dielectric constant, and Δρ/ρ is the change of resistivity.

The piezoresistive effect has been widely investigated and used in a broad range of fields, such as microelectromechanical systems and nanoelectromechanical systems. Various piezoresistive flexible sensors have been proposed to monitor the physiology of an individual owing to its ease of fabrication, simplicity in structure, cost effectiveness, capability for detection of static and dynamic signals, low power consumption, biocompatibility, and wide pressure-sensing range. However, this type of sensor suffers from low sensitivity for the detection of low pressure and power source dependency.

### 2.4. Triboelectric Mechanism

The discovery of the triboelectric effect began to emerge about 2600 years ago [64]. This effect involves the generation of electrical signals as a result of the surface contact between two triboelectric materials. When these materials are in contact with each other, charges will overlap and align according to polarity across the surface. When the applied stress is slowly removed, the contact electrification of the materials reduces, and a potential difference can be measured as a result of electrostatic induction. This phenomenon allows the charges to flow until a net equilibrium is achieved, thereby generating electrical signals [65]. The development of triboelectric nanogenerators in 2012 is one of the first studies that explored the triboelectric effect in sensors [66].

Meng and colleagues [67] employed an interlaced weaving approach to produce a triboelectric-based wearable sensor. The sensor was fabricated using the stacking layers of polyethylene terephthalate, indium–tin oxide, polytetrafluoroethylene, and PDMS. The developed sensor exhibited sensitivity of 45.7 mV Pa^−1^, response time of <5 ms, broad sensing range of ≈710 Pa, and LOD of 2.5 Pa. Another significant finding of this work is the low difference of 0.87–3.65% between BP readings obtained using the as-prepared sensor and the cuff BP monitor, depicting its potential as a self-powered wearable sensor for continuous BP monitoring.

The triboelectric sensor exhibits excellent properties for wearable applications, particularly physiological monitoring, due to its ability to self-power and detect dynamic signals, its light weight, and low fabrication cost. However, when this sensor is employed to monitor the vital signs of individuals, several issues, such as biocompatibility, biodegradability, and short service lifetime, occur.

Therefore, identifying the type of vital signs being monitored is crucial prior to the fabrication of flexible sensors. For instance, sensors developed in the form of patches are more suitable for continuous BP monitoring because they exhibit greater flexibility on the skin and are comfortable for long wear. Table 1 summarizes the advantages and disadvantages of different sensing mechanisms. Among them, the piezoresistive sensor has attracted much research attention in the field of health monitoring systems through wearable flexible sensors due to advantages including dynamic sensing, low cost and power consumption, and biocompatibility.

## 3. Sensing Performance Parameters

Sensing performance is another significant aspect of developing wearable flexible sensors. In this section, several key performance parameters of sensors are discussed. Sensitivity, or gauge factor (GF), defines the minimum input that is needed to create changes in electrical signals as a response to mechanical stimuli [68]. Limit of detection (LOD) describes the lowest pressure that can be detected by the sensor to generate electrical signals at the ground pressure of 0 Pa [69]. Response time is the time taken by a sensor to produce detectable changes in electrical signals in response to stress [55].

The stretchability of a sensor refers to its ability to retain internal conductive network connection and resist permanent deformation under external stress [70]. A sensor should maintain a trade-off between high sensitivity and good stretchability to ensure that the original device structure is maintained without neglecting subtle pressure detection under applied stress [71]. Linearity refers to the direct proportional relationship between electrical signals and mechanical stimuli within the set range limits. This parameter is represented by the coefficient of determination (*R*^2^), whereby the parameters are considered to achieve good linearity when the *R*^2^ value reaches 1 [63]. Detection range indicates the minimum and maximum pressure values that can be detected by the sensor.

These parameters should be considered during the device engineering phase to develop a flexible sensor for monitoring BP continuously and with great accuracy. High sensitivity, low LOD, fast response time, excellent stretchability, good linearity, and broad detection range are some of the desirable properties to realize wearable BP monitoring systems for future personalized medicine.

## 4. Two-Dimensional Nanomaterials for Flexible Sensors

Material selection plays an important role in developing a flexible sensor that can withstand mechanical stress and maintain its sensitivity without being permanently deformed. Functional materials that are suitable and safe for human consumption should be prioritized in the development of wearable flexible sensors due to the hemodynamic properties of the human arterial system. Aside from good mechanical flexibility, the developed flexible sensors should maintain electrical conductivity when subjected to deformation. Ag has the highest electrical conductivity among conductive materials with $6.3\times 10^{7}\;S\;m^{-1}$. Despite being widely used in a wide range of industrial applications, the high fabrication costs and tendency to electrical breakdown have been the major bottlenecks of Ag in the area of wearable flexible sensors. Meanwhile, Cu is viewed as a potential alternative to Ag due to its high electrical conductivity ($5.96\times 10^{7}\;S\;m^{-1}$) which is only 6% lower than Ag and cost effective. However, Cu is easily oxidized in ambient temperature, resulting in lower electrical conductivity and limiting its application in flexible sensors [72,73,74]. Consequently, there has been a surge of research into new materials for high-performance flexible sensors.

The greater competitiveness of 2D nanomaterials than their Ag and Cu counterparts has attracted tremendous interest to explore the versatility of these materials. Two-dimensional nanomaterials demonstrate outstanding properties in detecting subtle stimuli and enabling nanomaterial-based wearable electronics with high surface-area-to-volume ratio, electrical conductivity, mechanical flexibility, and light weight [75]. Moreover, 2D nanomaterials are excessively exploited for diverse studies, such as in biomedical engineering, environment, construction, and electronics [76,77,78,79]. This section summarizes the frequently used 2D nanomaterials for the fabrication of flexible sensors.

### 4.1. Graphene (Gr)

Gr is a class of 2D nanomaterials known as an allotrope of carbon and arranged in a hexagonal lattice structure [80,81,82]. Gr is a zero-band gap material with exceptional characteristics such as being ultrathin, light weight, and stronger than steel, and having high electron mobility [83,84,85]. Pristine Gr has the least crystalline defect, high purity, and thermal conductivity. Gr and its derivatives are in huge demand in multiple fields [86], such as in coatings [87], biosensors [88], batteries [89], and membranes [90]. Several attempts were conducted to modify the morphology of pristine Gr to tailor its practical applications through bandgap engineering [91]. For instance, numerous Gr-based flexible sensors have been developed recently.

**Figure 2:**
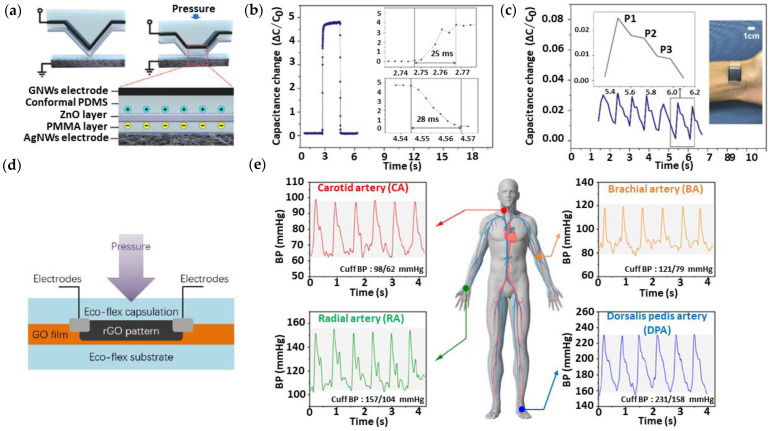
Schematic, (**a**) response time, (**b**) and real-time pulse wave detection from the time-resolved capacitance change response (**c**) of the piezocapacitive GNWs/PDMS/ZnO sensor. Reproduced with permission from [94]. Copyright Elsevier, 2021. (**d**) Cross-section of the proposed sensor, and (**e**) multiple pulse waveforms measured at different locations. Reproduced with permission from [95]. Copyright American Chemical Society, 2020.

Due et al. [92] successfully fabricated a wearable piezoresistive sensor by facile preparation methods. Gr was grown on a non-woven fabric (GNWF) substrate to produce a wearable sensor that is compatible with cloth. The sensor had excellent sensitivity of around 0.057 kPa^−1^ and GF of −7.1 at the applied strain of 1% for 10,000 cycles. Ai et al. [93] demonstrated a piezoresistive sensor based on reduced graphene oxide (rGO) sandwiched in between PDMS films. This sensor was tested on a healthy subject and successfully detected pulse waveforms, including the diastolic tail which the tonometry method failed to sense. The results showed that this flexible sensor can be further extended into health wearables to monitor BP continuously with 50.9 kPa^−1^ sensitivity, 3 Pa LOD, ~1 µW power consumption, 50 ms response time, and high durability. Based on Figure 2a, Luo et al. [94] presented a piezocapacitive sensor that was micro-fabricated with graphene nanowalls (GNW), PDMS, and zinc oxide (ZnO) to monitor pulse for wearable applications. Figure 2b,c depict that the proposed technique exhibited improved sensing performance with 22.3 kPa^−1^ sensitivity, 25 ms response time, and 22 kPa sensing range. Wu et al. [95] developed a Gr-based piezoresistive sensor by using a laser scribing method for monitoring BP, as shown in Figure 2d. The sensor showed an average sensitivity of 12.3 kPa^−1^ and enabled different BP measurement locations (Figure 2e), which is advantageous for non-invasive monitoring.

Gr has the disadvantages of high fabrication cost and lack of well-established synthesis methodologies, contributing to the challenging preparation of a monolayer with excellent quality, controllable thickness, and mass-produced Gr [96]. Despite the continuous switching behavior of Gr, making it difficult for energy storage devices, this problem can be addressed by opening the bandgap with regard to the favorable on–off switching properties [97].

### 4.2. Transition Metal Dichalcogenides (TMD)

The discovery of other 2D nanomaterials has become more popular since the successful findings in 2004. TMDs have gained increased attention for 2D nanomaterials because their crystal structures are similar to those of graphite. The acronym of TMDs is derived from MX_2_, where M describes the transition metal from groups 4 to 11, such as molybdenum (Mo) and stannum (Sn). Meanwhile, X represents the elements of group 16, such as sulfur (S) and selenium (Se), which are known as chalcogens [85]. The heterostructure engineering of TMDs is possible due to their weak van der Waals interlayer interaction and easily tunable bandgap, which are not found in Gr [98,99]. TMDs exhibit good electronic and optical properties and are suitable for biosensors [100], light-emitting diodes [101], and memory devices [102]. Besides the widely used Gr, progress has been achieved in developing flexible sensors based on TMDs for designing biomedical devices.

**Figure 3:**
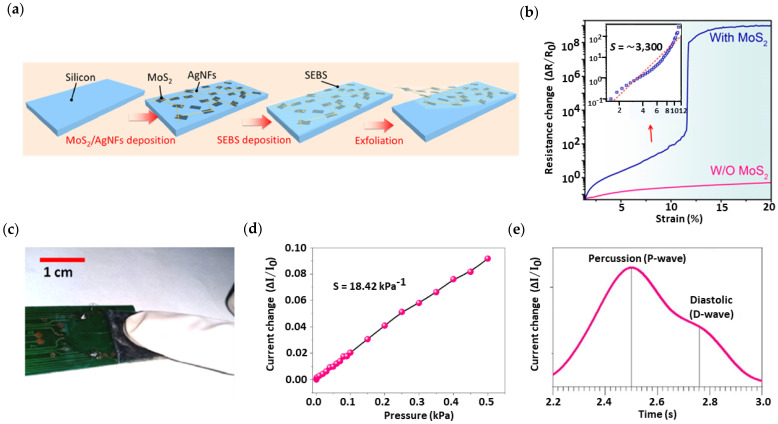
(**a**) Schematic illustration of the fabrication process. (**b**) Relative resistance change–strain curves of the MoS_2_/Ag nanofiber strain sensor with GF of 3300 in the range between 1.4% and 11.5%. Reproduced with permission from [103]. Copyright American Chemical Society, 2019. (**c**) Photographic image of the piezoresistive MoS_2_/cellulose paper sensor under fingertip loading. (**d**) Sensing response of the sensor for pressure range from 0.001 to 0.5 kPa. (**e**) Pulse wave acquisition in real-time using the proposed pressure sensor with a peak-to-peak time difference of 260 ms. Reproduced with permission from [105]. Copyright Elsevier, 2021.

For instance, Qiu et al. [103] proposed a piezoelectric MoS_2_ sensor by forming conductive networks between MoS_2_ nanosheets and Ag nanofibers in an elastic conductive film. Figure 3a,b show that the developed sensor possessed significant potential in health wearables due to its ability to detect systolic and diastolic peaks, a sensitivity of 3, 3000, 0–13% strain range, and relatively fast response of ∼850 ms for stretching and relaxation time. Furthermore, Tannarana et al. [104] reported a wearable multimodal sensor developed from SnSe_2_ nanosheets grown on paper substrate through liquid-phase exfoliation. The sensor exhibited excellent sensing performance with 868% responsivity, 1.79 kPa^−1^, 100 ms response time, 2–100 kPa detection range, and 5000 cycles of loading pressure. Moreover, Pataniya et al. [105] manufactured a flexible piezoresistive sensor based on MoS_2_ nanosheets by using a cellulose paper as substrate, as shown in Figure 3c. The sensor had high sensitivity of 18.42 kPa^−1^ (Figure 3d) for the low-pressure range, sensing range of 0.001–0.5 kPa, and fast response time. The time difference displayed in Figure 3e between systolic and diastolic peaks measured was 260 ms, which is in agreement with previous studies.

TMDs have shown potential for flexible sensors to monitor vital signs; however, the current synthesis techniques are not yet scalable or reliable for producing monolayers that are defect-free and have controllable thickness and excellent quality [106,107].

### 4.3. Transition Metal Carbides and Nitrides (MXene)

Gogotsi and his team [108] conducted the first experiment on MXenes in 2011 by using hydrofluoric acid (HF) to exfoliate three-dimensional titanium aluminum (Al) carbide ($M_{n+1}{\mathrm{AlX}}_{n}$) and produce layers of 2D titanium carbide ($M_{n+1}X_{n}T_{x}$). M represents transition metals, such as Mo, titanium (Ti), and niobium (Nb); X denotes carbon/nitrogen; and T refers to functional termination groups, such as hydroxyl (-OH), fluorine (-F), and oxygen (-O) [109]. As the name reflects the elements, these materials comprise multi-layers of transition metal carbides, nitrides, and carbonitrides [110]. Recently, MXene has been found to be an ideal candidate for a wide range of applications, such as batteries [111], conductive fillers [112], supercapacitors [113], and solar cells [114].

A growing trend on the development of MXenes-based wearable sensors has occurred due to their superior electrical conductivity, peculiar multilayer structure, and large surface-area-to-volume ratio [115]. For instance, the abundant functional termination groups and the hydrophilic surface in MXenes’ nanostructures confer them with great capability to mix with polymers. The interlayer spacing in between MXene multilayers and the hybridization with polymers in flexible devices would significantly enhance sensor performance including enhanced sensitivity and greater electrical conductivity [116,117].

**Figure 4:**
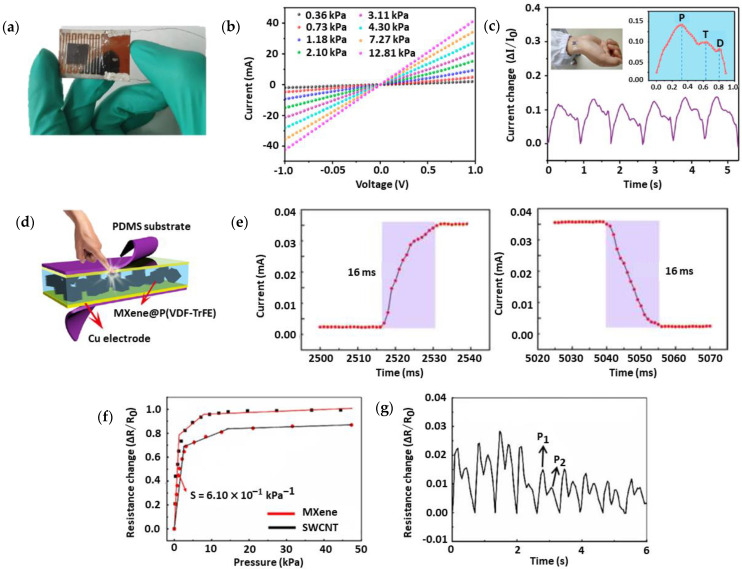
(**a**) Photographic image of the piezoresistive Ti_3_C_2_T_x_/PDMS pressure sensor. (**b**) Linear current–voltage characteristics indicate the piezoresistive performance of the sensor. (**c**) Real-time detection of subtle radial pulse signals using the fabricated sensor. Reproduced with permission from [118]. Copyright American Chemical Society, 2020. (**d**) Schematic representation of Ti_3_C_2_T_x_/P(VDF-TrFE) flexible pressure sensor with dimensions of 10 mm by 8 mm. (**e**) Fast response/recovery time demonstrated by the sensor in 16 ms. Reproduced with permission from [119]. Copyright American Chemical Society, 2020. (**f**) Pressure sensitivity of the Ti_3_C_2_T_x_/PDMS flexible sensor of 0.61 kPa^−1^ is defined by the slope of the relative resistance change–pressure curve. (**g**) Real−time radial pulse monitoring using the developed sensor. Reproduced with permission from [120]. Copyright Elsevier, 2021.

Figure 4a,b show a novel flexible piezoresistive sensor based on Ti_3_C_2_T_x_/PDMS developed by Cheng et al. [118] using simple abrasive paper stencil printing to detect the pulse wave of an individual. The sensor detected three important peaks of pulse waveform (Figure 4c) and achieved sensitivity of 151.4 kPa^−1^, response time of <130 ms, and LOD of 4.4 Pa. The sensor’s response showed high competency in continuous health monitoring. Li et al. [119] developed a Ti_3_C_2_T_x_ flexible piezoresistive sensor on poly (vinylidene fluoride) trifluoroethylene (P(VDF-TrFE)) substrate by spin coating, as shown in Figure 4d. The prepared sensor successfully detected the pulse wave peaks and exhibited excellent properties, with 817.4 kPa^−1^ sensitivity, 16 ms response time (Figure 4e), and 0.072–0.74 Pa detection range. Su et al. [120] fabricated a flexible piezoresistive sensor based on Ti_3_C_2_T_x_ by using the honeycomb approach on PDMS conductive film. Figure 4f shows that the sensor had a sensitivity of 0.61 kPa^−1^, response time of 160 ms, and detection range of 0–50 kPa. Not only that, but radial pulse waveforms were also detected in real-time using this sensor, as shown in Figure 4g.

Although MXene can be regarded as an ideal candidate for flexible sensing applications, it has several disadvantages that can affect its long-term stability. MXene films have greater tendency to experience fracture due to poor mechanical properties caused by the weak van der Waals interaction in MXene nanosheets [121,122]. Moreover, the exfoliation of the Al layer from the MAX phase by using HF etchant may introduce nanosheet restacking, leading to easy oxidation in the ambient environment and reduced biocompatibility [123].

In summary, 2D nanomaterials exhibit potential as functional materials in flexible sensors due to their large surface-area-to-volume ratio, which leads to high sensitivity, fast response time, and wide detection range. Although a number of studies have reported on flexible sensors utilizing these nanomaterials, well-grounded conceptual and methodological approaches for continuous BP monitoring are lacking. The majority of these studies on wearable applications have focused only on human activity detection. Few studies have attempted to validate the BP readings obtained from the proposed sensors with those from a commercial cuff sphygmomanometer. The findings reported by previous studies (Table 2) provide insights for future research and better understanding on this matter.

## 5. Wearable Blood Pressure Monitoring

**Figure 5:**
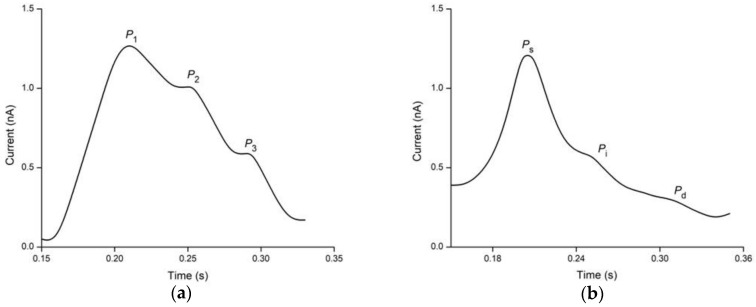
Common BP pulse waveform shapes and pulse peaks. (**a**) Radial pulse is measured from the radial artery of the wrist. (**b**) Carotid pulse is measured from the carotid artery of the neck.

BP is an essential vital sign in human health monitoring, and its complex dynamics make it difficult to understand. Based on the American Heart Association, the normal systolic BP (SBP) and diastolic BP (DBP) of healthy people are ≤120 and 80 mmHg, respectively [124]. Individuals with BP readings beyond the normal range are considered as hypertensive [125], while those with readings lower than the baseline are considered normotensive [126]. A BP pulse waveform consists of three main peaks, namely, percussion wave (P wave), tidal wave (T wave), and diastolic wave (D wave). In general, P wave is the early SBP, T wave is the late SBP, and D wave is the DBP [127]. Figure 5 depicts the typical BP pulse waveforms obtained from different measurement sites (wrist and neck) of an individual. According to Figure 5a, these waves can be observed from the radial artery pulse and are denoted by $P_{1}$ (P wave), $P_{2}$ (T wave), and $P_{3}$ (D wave), respectively. With the carotid artery pulse in Figure 5b, three distinct peaks can be extracted with the systolic peak denoted by $P_{s}$, $P_{i}$ is the inflection point and $P_{d}$ is the diastolic peak.

Arterial catheterization is the gold standard for continuous measurement of BP, yet it is invasive and widely employed only on critically ill patients. Meanwhile, sphygmomanometers can monitor BP unobtrusively but suffer from several limitations, such as intermittent readings and non-portability. In this regard, wearable flexible sensors are expected to pave new routes toward remote health monitoring as the future of telemedicine. This section presents several examples of cutting-edge wearable technologies for non-invasive and continuous monitoring of BP.

Noh et al. [128] integrated four components, namely, ECG, ballistocardiogram (BCG), flexible electronic circuits, and a ferroelectric film, in a sensing platform. Silver paste was used to sandwich the ferroelectric layer in between the ECG and BCG through a screen-printing method. Three healthy subjects were selected to participate in the study. Simultaneous measurements were conducted using the developed sensor and a reference device, Finapres. The sensor showed comparable SBP values from both devices with the mean error and standard deviation of −0.16 and 4.12 mmHg, respectively. Although this work did not measure DBP, the results were promising and proved the feasibility of a flexible BP patch as an alternative to the current sphygmomanometer for continuous BP monitoring.

Dagdeviren et al. [129] fabricated a piezoelectric pressure sensor patch by using PZT and Si substrate for long-term BP monitoring. Here, the design of the sensor played a significant role as the PZT is more susceptible to deformation than the Si substrate, allowing the sensor to be skin-mounted. The average SBP and DBP readings of three volunteers recorded using this device are in agreement with the cuff BP values, with averages of 110 mmHg and 65 mmHg for SBP and DBP, respectively. The sensor can stretch up to 30% of its size and has 0.1 ms response time, 25 µm thickness, and 0.005 Pa LOD. The developed sensor can be beneficial for continuous monitoring of human health. Luo et al. [130] documented a skin patch that can monitor continuous BP non-invasively. The sensing mechanism is based on the piezoresistive properties of carbon-made textile and epidermal ECG sensors. Compared with the cuff BP monitor, the fabricated sensor had small deviations of 6.5 ± 4.9 mmHg and −0.4 ± 3.9 mmHg for SBP and DBP, respectively, as well as low power consumption of 3 nW. Hence, the sensor has potential to be integrated in health wearables.

Wang et al. [131] reported an ultrasonic wearable sensor patch for continuous monitoring of BP. A piezoelectric composite and a thin Si layer were used as the sensing element and elastomer, respectively. Ultrasonic waves were employed to provide deeper penetration for BP detection. The proposed sensor had remarkable results and exhibited superior properties over tonometry modality for long-term monitoring of BP with up to 60% stretchability, 23.6 mW power consumption, and ~5 Pa loading pressure on the skin. Sempionatto et al. [132] extended the work by Wang et al. by developing ultrasonic electrochemical sensors based on PZT and printed polymer composites for monitoring BP and other physiological parameters, such as heart rate (HR), glucose, and lactate. Participants were given different stimuli, including food and drink, as well as physical activities, before and after measurement of the parameters. The developed epidermal sensor patch exhibited satisfactory performance when validated with cuff BP readings, thus demonstrating its suitability as a multimodal sensing device for wearable BP application. All studies involving wearable flexible sensor patches for monitoring continuous BP reported no negative effects, such as allergic reaction, redness, or damage, after the removal of the patches from the skin.

Recently, Kireev et al. [133] developed a piezoresistive e-tattoo for continuous BP monitoring by transferring Gr onto a tattoo paper using polymethyl methacrylate. Compared to earlier studies, BP accuracy was improved with 0.2 ± 5.8 mmHg and 0.2 ± 4.5 mmHg, for SBP and DBP, indicating good biocompatibility and stable BP measurement. Laurila et al. [15] proposed inkjet and blade-coating fabrication methods for developing an e-tattoo sensor with piezoelectric properties. The fabricated device is the thinnest fully printed piezoelectric sensor with 4.2 µm thickness. The sensor showed an improved sensitivity of ~50 times that of the reference BP monitor that exploits the arterial tonometry principle. Flexible sensors with e-tattoo configurations were developed in both studies due to the advantages of measuring continuous BP non-intrusively while maintaining good biocompatibility. For example, sensor fabrication on tattoo paper substrates improved skin adhesion and user experience for long-term wear. Moreover, the biomedical company Vivalink introduced the first commercial multi-sensing patch to monitor BP continuously and remotely [134]. The sensor detected various vital signs such as SBP, DBP, and HR with the universal regulatory clearance. This technology features small design, wireless connection, real-time monitoring, and water resistance.

Long-term remote BP monitoring is feasible and relatively accurate with the emergence of advanced technologies and 2D nanomaterials. The ground-breaking research efforts are significant for the early diagnosis and prognosis of health complications such as cardiovascular diseases (CVD). The detection of CVD continues to be a challenge for physicians, and late-stage diagnosis often leads to stroke incidence and mortality. Continuous BP monitoring using wearables, such as epidermal patches and e-tattoos, enables timely treatment for high-risk patients, particularly those with hypertension as it is an underlying risk factor of CVD. This monitoring system is expected to facilitate real-time BP monitoring through automated notification of abnormal BP readings, triaging perioperative patients, and mitigating motion artifacts in other cuffless BP monitors.

## 6. Challenges and Future Outlooks

Despite the successful development of laboratory-based cuffless sensors for continuous BP monitoring, further improvements are required to address their limitations prior to reaching market readiness and clinical acceptability. Thus far, the synthesis of 2D nanomaterials remains a major challenge in wearable sensing implementation. Existing techniques such as exfoliation suffer from weaknesses that hinder their abilities to fabricate a high-quality and zero-defect sensing layer. To address this issue, development of suitable 2D nanomaterials with the benefits of low fabrication cost and mass-production is the potential area of focus for future research.

Another key challenge for the implementation of wearable is biodegradability, which is a growing health concern worldwide. For instance, the use of toxic chemicals, such as HF acid, to produce 2D nanomaterials can cause adverse effects on the environment and humans through the release of chemical waste and the emission of poisonous gases. Therefore, continuous research efforts are needed to fabricate wearable sensors based on 2D nanomaterials that contain non-harmful by-products and are safe for humans.

Currently, it remains challenging to realize a fully wearable flexible sensor and the development is constrained by the power source dependency. The developed prototypes still heavily rely on wired connection for continuous power supply and signal transmission. A microcontroller-based flexible sensor has been suggested to overcome this problem, yet it is not practical for everyday use and has low conformability. Hence, future research should incorporate the integration of on-chip circuits in wearable devices to be self-powered for monitoring BP and other vital signs continuously and highly accurately. In addition, inkjet printing of electronics in flexible sensors is gaining much research attention in regard to the development of fully integrated wearable technologies.

Monitoring of BP is technically challenging because it involves a manual handling task. In the future, the potential use of artificial intelligence (AI) should be explored because it is at the forefront of smart wearables for continuous physiological monitoring. Aspects that should be investigated are the integration of machine learning (ML), as a subset of AI, to enhance the performance of wearable sensors through the prediction of vital signs, the generation of precise outputs, and real-time monitoring and seamless transmission of signals. Furthermore, the adoption of ML in wearable technologies offers model personalization through user behavior learning.

Despite the potential of 2D nanomaterials in flexible sensors, their commercial applications to wearable BP devices are still far from being achieved. One of the challenges is the stability of 2D nanomaterials when integrated into wearable technologies, leading to difficulty in large-scale production. This problem has inspired scholars to explore the potential solutions through available and emerging techniques for upscaling of 2D nanomaterials synthesis and their integration on wearable platforms with improved sensing performance.

## 7. Conclusions

In this review, we summarize the recent development of wearable flexible sensors for BP monitoring. Common transduction mechanisms such as piezoelectric, capacitive, piezoresistive, and triboelectric were introduced, and performance parameters including sensitivity, response time, detection limit, stretchability, and biocompatibility were discussed. Moreover, 2D nanomaterials, such as Gr, TMDs, and MXenes, emerged as promising candidates for flexible sensors for BP monitoring. The comparison between novel 2D nanomaterial-based flexible sensors and their respective sensing performance were presented. Several examples of wearable flexible sensors for BP monitoring were also reviewed. This review aims to add to a growing body of literature on the development of wearable sensing systems based on 2D nanomaterials for unobtrusive, continuous, accurate, and reliable BP monitoring.

## Figures and Tables

**Table 1:** Advantages and disadvantages of flexible sensors based on different transduction mechanisms: piezoelectric, capacitive, piezoresistive, and triboelectric.

Type	Advantage	Disadvantage
Piezoelectric	Dynamic sensing Quick response time Low power consumption	Unable for static sensing Less sensitive for low-pressure sensing Harmful lead-based material
Capacitive	Highly sensitive for low-pressure sensing Low power consumption Wide detection range	Slow response time Expensive Power source dependent
Piezoresistive	Ease of fabrication Simple structure Cost-effective Static and dynamic sensing Low power consumption Biocompatibility Wide detection range	Less sensitive for low-pressure sensing Power source dependent
Triboelectric	Power source independent Dynamic sensing Lightweight Cost-effective	Biocompatibility Biodegradability Short service lifetime

**Table 2:** Comparison of flexible sensors based on 2D nanomaterials for BP monitoring.

Materials	Sensing Principles	Sensitivity/ GF	Response Time (ms)	Ref.
GNWF	R *	0.057 kPa^−1^	-	[92]
rGO/PDMS	R	50.9 kPa^−1^	50	[93]
GNWs/PDMS/ZnO	C **	22.3 kPa^−1^	25	[94]
Gr/Eco-flex	R	12.3 kPa^−1^	-	[95]
MoS_2_/Ag nanofiber	E ***	3300	850	[103]
SnSe_2_/Paper	R	1.79 kPa^−1^	100	[104]
MoS_2_/Cellulose paper	R	18.42 kPa^−1^	260	[105]
Ti_3_C_2_T_x_/PDMS	R	151.4 kPa^−1^	<130	[118]
Ti_3_C_2_T_x_/P(VDF-TrFE)	R	817.4 kPa^−1^	16	[119]
Ti_3_C_2_T_x_/PDMS	R	0.61 kPa^−1^	160	[120]

* R = piezoresistive. ** C = capacitive. *** E = piezoelectric.

## Data Availability

Not applicable.
